# New Appliances and Things Medical

**Published:** 1896-03-14

**Authors:** 


### NEW APPLIANCES AND THINGS MEDICAL.

[We shall be glad to receive, at our Office, 428, Strand, London, W.C., from the manufacturers, specimens of all new preparations and appliances, which may be brought out from time to time.]

### CEREBOS SALT COMPANY, NEWCASTLE-ON-TYNE.

In a previous number of The Hospital we gave an analytical account of this salt. The preparation consists of a table salt corresponding to the natural salt existing in vegetables, and is therefore not only a flavouring but a constituent of wholesome food. From recent samples supplied to us we find that it maintains its high standard, and, as it is sold at a reasonable price, will no doubt increase in popularity as it is better known.

### ITALIAN BURGUNDY.

Methley and Co. (Limited), 150, Fenchurch Street, E.C.

Messrs. Methley and Co. have submitted to us a sample of their Italian Burgundy, which they guarantee as a pure natural wine. The samples which we have tested are agreeable in taste, and the wine is of fine colour. Our analyst finds the total acidity to amount to 3·15 per cent., of which 0·27 per cent. is mineral matter. The alcoholic strength is 11·47. The acidity mentioned is equivalent to 78·5 c.c. of decinormal soda per 100 c.c. of the wine, the volatile acidity being 25·5 c.c. The sample contained 0·74 per cent. of sugar. It will be seen from a comparison of the analyses of other samples of Italian Burgundy which we have analysed, that this particular sample is remarkable for its high alcoholic strength. We think the wine will prove beneficial to patients when recovering from illness.

### LIQUEUR WHISKY.

(W. Stenhouse and Co., Glasgow.)

This whisky is now finding much favour with the profession, who realise that brandy is not the only spirituous stimulant suitable for private or hospital practice. Indeed many patients have an aversion to brandy in all its forms. A pure and matured whisky as that of the above firm is most acceptable to many patients, and from the report of medical men who have prescribed it, the therapeutic effect has been all that could be desired.

### ALUMINIUM UMBILICAL PADS.

(Reynolds and Branson, 13, Briggate Street, Leeds.)

This little appliance is in shape something like a soup-plate, and of about the size of a two-shilling piece; the flange is perforated by four small holes, so that it can be sewn on to the baby's body-belt, and so kept accurately in an inverted position over the umbilical stump. Another plan for keeping the pad in position is to apply strips of strapping. The advantages claimed for this little invention are—firstly, the cheapness (4s. per dozen); secondly, the lightness; and thirdly, the cleanliness. These pads by very reason of their simplicity have much to recommend them, and doubtless they will meet with a good reception in the nursery.

